# Palliative care training programmes for community volunteers working with children and their families: a scoping review

**DOI:** 10.3389/fpubh.2025.1469854

**Published:** 2025-05-16

**Authors:** Nattaporn Ontrakrai, Cara Bailey, Tracey Valler, Susan Neilson

**Affiliations:** ^1^School of Nursing and Midwifery, Institute of Clinical Sciences, University of Birmingham, Birmingham, United Kingdom; ^2^Department of Pediatric Nursing, Faculty of Nursing, Chiang Mai University, Chiang Mai, Thailand; ^3^St Giles Hospice, Lichfield, United Kingdom

**Keywords:** palliative care, end-of-life care, child, volunteers, education

## Abstract

**Introduction:**

Volunteers play a significant role in enhancing the quality of palliative care. Training is necessary to deliver voluntary community care that assists healthcare professionals, benefits families and eradicates inequities for underserved populations. Community volunteers form a large part of paediatric palliative care support, but training courses are rarely specific to children leaving volunteers unprepared and unsupported.

**Aim:**

To identify existing literature and synthesise knowledge gaps related to volunteer training in community paediatric palliative care.

**Method:**

This scoping review was guided by Arksey and O'Malley and Levac et al. We conducted searches for published literature on palliative care education published between 2000 and 2023 through comprehensive searches of the Web of Science, CINAHL Plus, MEDLINE, PsycINFO, Scopus, and Cochrane Library databases.

**Results:**

In total, 16 (out of 590) articles met the eligibility criteria and were extracted for review. No articles exploring paediatric palliative care training for community volunteers were identified but findings from research with adults suggest that training should be tailored to volunteers' responsibilities, local needs, and culture. Training is varied but volunteers will likely benefit from blended learning that engages with communities. Four predominant domains were identified: public health palliative care education, factors related to developing community volunteers, main topics and training contents, and training models for community volunteers.

**Conclusions:**

Globally, the provision of palliative and end-of-life care is increasingly falling to carers, volunteers, and public health workers. Education is vital to prepare volunteers, improve confidence and offer support. This first scoping review of volunteer training provides much-needed evidence to guide future educational development for the informal workforce and identifies a gap for original research specific to paediatrics.

## 1 Introduction

Palliative care is a crucial component of the care process that aims to improve or maintain the quality of life for individuals with serious or life-limiting illnesses (1). Providing palliative care for children is proactive and dynamic. It focuses on continuous care from diagnosis to end-of-life care and also follows up after the child's death (2). Globally, around 2.5 million children, who have experienced serious health-related suffering, are dying each year (3). Approximately 21 million children throughout the world are considered to need palliative care each year (4). In particular, over 97% of those children are located in low- and middle-income countries (5). Life-threatening illnesses have a substantial impact on children's physical, emotional, and psychological wellbeing, resulting in a reduction in their quality of life.

The provision of palliative care varies in different countries around the world. Approximately 14% of the world's population can access advanced integrated palliative care provision, which is the comprehensive care service found in many European countries (6). Several studies report challenges, particularly in low- and middle-income countries, such as the need for greater readiness for integrated care, accessibility of services and education and workforce development (7–9). Furthermore, local palliative care services for children are restricted due to a primary focus on adult patients, a shortage of resources or equipment, and insufficient training for healthcare workers (7, 10). These challenges highlight the importance of the public health approach alongside the statutory provision of palliative care.

Within the public health approach to palliative care, a compassionate community refers to asset-based community engagement in addition to pre-existing professional care, recognising that end of life care is everyone's responsibility (11). Support from a patient's inner network, such as family, friends, neighbours, or co-workers, as well as from their outer network, which may be loosely connected such as community volunteers, can alleviate suffering (12). For instance, the act of simply listening can bring a lot of relief and comfort to people who are feeling stressed. Engaging people in the community is essential to reducing social morbidities caused by long-standing illnesses (11). Abel et al. (13) point out that establishing community health services, which are connected to primary care, can significantly reduce unplanned hospital admissions. Increasing evidence suggests that the involvement of communities in addressing healthcare problems leads to more effective results than when it is left solely to professionals (14–16). Thus, a critical aspect of this integration is the effective education of volunteers, who form part of the informal workforce in palliative care services.

A scoping review (17) indicates that most interventions related to compassionate communities are reported to improve individual competency by delivering training and enhancing awareness programmes. Moreover, the Last Aid Course has been deployed with non-professionals using the concept of teaching at a public level in several European nations. The curriculum demonstrated significant improvements in palliative care understanding (18). However, many studies have analysed training courses designed for medical staff and carers, but evidence on curriculum design for volunteers, particularly in terms of children's palliative care, is sparse (19–21). Volunteer training in palliative care has gained attention in recent years as the community-based palliative care model has expanded. The lack of paediatric palliative care education for volunteers is a significant challenge that impedes the improvement of paediatric palliative care networks in the community.

Thus, a scoping review was considered the most appropriate approach, as it aims to map existing evidence and synthesise knowledge gaps in developing paediatric palliative care training for community volunteers. We aim to explore palliative care training programmes for community volunteers, identify factors related to the development of volunteers' skills, map the learning contents, and identify the learning models for volunteers in communities. This information may be helpful in light of the increasing development of palliative care education for volunteers to care for children receiving palliative care and their families.

## 2 Methods

A scoping review is a type of evidence review that synthesises available information from a variety of sources using a systematic method (22). Such reviews aim to explore the breadth of a certain subject, which can help to identify the main characteristics of the literature, clarify concepts, investigate research conducted, provide preliminary results for a systematic review, or identify knowledge gaps (23). This scoping review was informed by the framework developed by Arksey and O'Malley (24) and additional recommendations by Levac et al. (25), which improve clarity and transparency during the review process. The review consisted of five main steps: identifying the research question, identifying relevant studies, selecting studies, charting the data, and reporting the results. For this study, the optional sixth stage—consulting with stakeholders—was not implemented. The focus of this review was to map all relevant evidence on volunteer training in palliative care.

### 2.1 Identifying the research question

The first step of this scoping review of the literature was guided by the research question: “what is known about training programmes for volunteers providing paediatric palliative care in the community?”

### 2.2 Identifying relevant studies

The search strategy for the related literature was augmented using guidelines from the Joanna Briggs Institute (JBI), which recommends using the population, concept, context (PCC) framework to identify key concepts in the review questions, the literature search, and the inclusion criteria (26). Search terms and associated descriptors of paediatric palliative care training programmes for community volunteers were used to search for relevant evidence by consulting a research librarian from University of Birmingham. An extensive search was performed using the Web of Science, CINAHL Plus, MEDLINE, PsycINFO, Scopus, and Cochrane Library databases. The search retrieved book chapters, review articles, and research articles (qualitative, quantitative, and mixed methods) published in peer-reviewed journals. Citation searches in relevant topics were also used to aid for comprehensiveness. Keywords and MeSH headings were used in the search, and the Boolean operators were mixed with the search keywords. See Supplementary material S1 for the search string used for the MEDLINE database.

The search was first conducted in February 2023 and was rerun using the original search strategy on the 13 January 2024 to identify additional articles that may have been published since the initial search. We included studies that were published in the English language from 1 January 2000 to 31 December 2023. The foundational concepts of compassionate communities started to receive recognition in the field of palliative care from the early 2000 (11). Thus, this review searched for studies published from 2000 onward to assemble a more current and comprehensive list of relevant publications. The search data were transferred to Rayyan, an online systematic review software, for screening. A total of 588 articles were generated from the search strings **(**CINAHL Plus**:** *n* = 127; Web of Science: *n* = 70; Cochrane Library: *n* = 53; Scopus: *n* = 139; MEDLINE: *n* = 184; PsycINFO**:** *n* = 15), and two additional articles were included through citation searching (total *n* = 590). After any duplicate studies were removed, 360 articles were available for screening.

### 2.3 Inclusion and exclusion criteria for study selection

Articles were screened against the inclusion and exclusion criteria (Table 1). All titles and abstracts were independently screened by two reviewers; NO screened all, and SN, CB, and TV each screened one third. Any conflicts that occurred during the screening steps between the two reviewers were flagged and resolved through group discussion until a consensus was reached. This resulted in a total of 29 articles for the full-text review.

**Table 1:** Inclusion and exclusion criteria.

**Criteria**	**Inclusion**	**Exclusion**
Population	- Studies on volunteers and citizens are included.	- Family members.
Concept	-Studies focused on palliative care training programmes for volunteers with children and young people, including those caring for adults.	-Studies focused on training healthcare professionals are excluded.
Context	-The provision of care in the community and in rural areas in all countries is included.	-Hospices and hospitals are excluded
Study design	- All types of studies published in English are included. - Book chapters, review articles, and research articles (qualitative, quantitative, or mixed-methods) published in peer-reviewed journals.	- Abstract-only publications (i.e., no full-text article available), theses and dissertations. - Grey literature. - Systematically constructed reviews were not analysed directly but were used to identify additional primary studies that met the inclusion criteria.
Time period	During 2000–2023	

**Figure 1:**
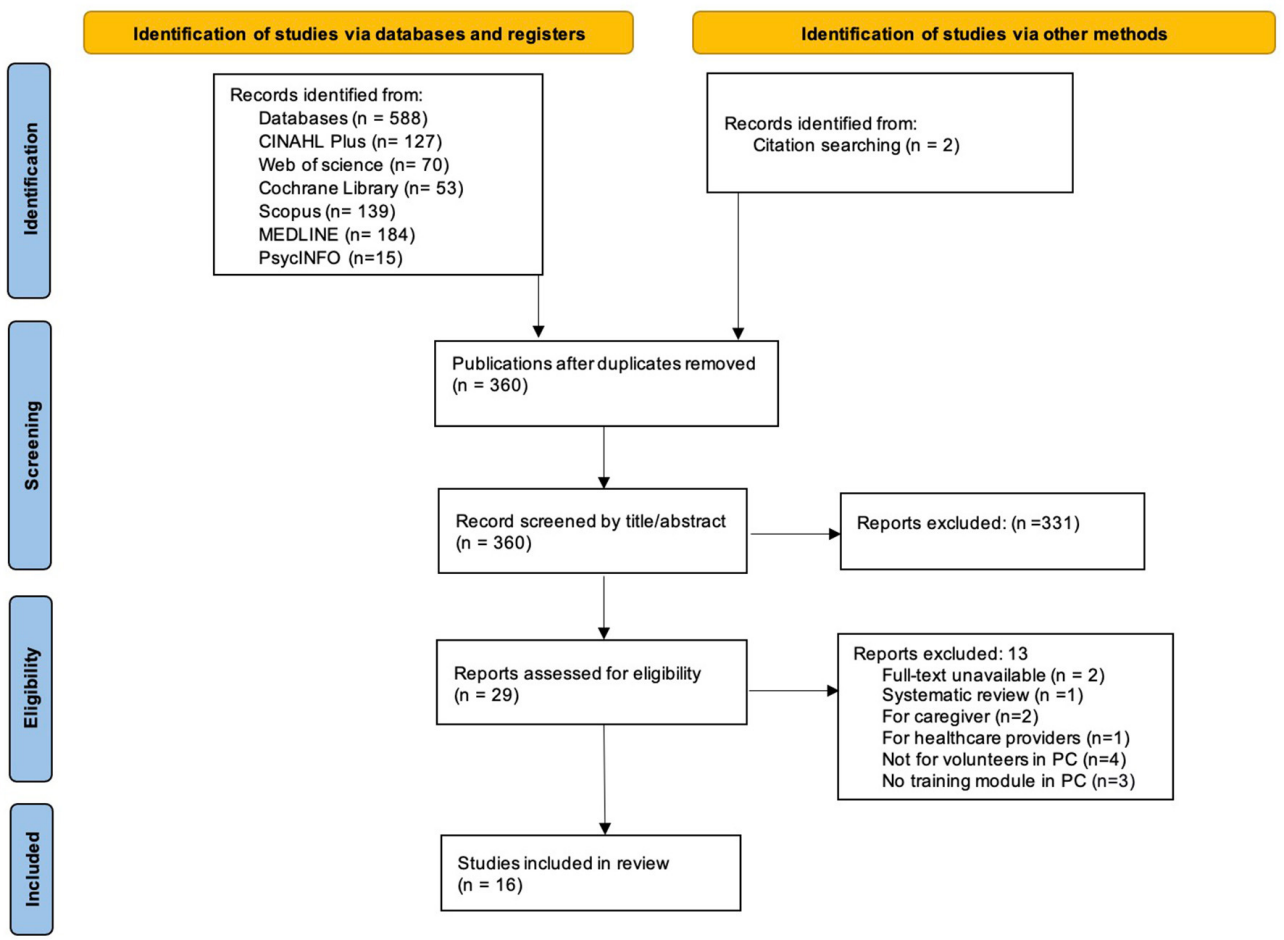
PRISMA flow chart of the study selection process.

Based on the eligibility criteria, the full-text screening was completed by NO and then discussed with team members to clarify details. The relevant studies that met the inclusion criteria were downloaded as full text and exported to referencing software (Mendeley). This yielded 16 full articles for inclusion in the review (Figure 1).

### 2.4 Charting and summarising the data

We followed the framework provided by Arksey and O'Malley, which guided the extraction of data using a charting template to describe and summarise information. The extracted data included the author(s), year of publication, study location, study type, aim, study population, methodology and methods, outcomes and/or important results (Table 2 see Supplementary material S2). The details and key findings were summarised in tables, which were collaboratively examined for accuracy by all of the team members. To identify thematic areas across the included studies, the extracted data were used to collate and summarise the findings using a content analysis approach (27, 28).

## 3 Results

Of 590 identified, articles covering both adults and children, 16 met our eligibility criteria. Eleven studies (29–39) discussed palliative care training in general, while four (40–43) focused on adult patients. Only one study (44) concentrated on paediatric palliative care.

### 3.1 Characteristics of the included studies

The 16 articles included in the scoping review were published between 2007 and 2023. Among them, 81.25% were published within the last decade (2014–2023) and 56.25% within the last 5 years (2019–2023). The studies varied in type; six had mixed-method research designs (30, 31, 39, 41–43) and five were qualitative (32, 34–36, 44). Of the five qualitative studies, four comprised action research and one took the form of a descriptive phenomenological study. Another two studies used quantitative research (33, 37). The remaining three studies took the form of a descriptive study (38), a review article (29), and study protocol (40). The included articles encompassed six studies in America (32–34, 36, 39, 42), five in Europe (30, 35, 37, 38, 44), two in Asia (41, 43), one in Australia (40), and one (31) that examined multiple regions (Germany and Brazil). Only one review article (29) did not specify the study location.

### 3.2 Review findings

Conceptual similarities in the data were grouped according to common themes. The review identified four major themes concerning available palliative care volunteer training: public health palliative care education, factors related to developing community volunteers, main topics and training contents, and training models for community volunteers.

#### 3.2.1 Theme 1: public health palliative care education

Public health is a science involving a multidisciplinary field that promotes wellbeing, prevents diseases, and improves the health outcomes of people and communities. Public health education leads to addressing gaps in palliative care knowledge and supporting healthcare systems. Of the studies reviewed, three (29–31) aimed to educate general citizens on palliative care knowledge; three (32, 34, 36) emphasised problem solving based on community needs and strengthening community development through training health professionals, volunteers, carers, and community members; and nine (33, 35, 37–43) studies explored the development of volunteers within palliative care or community services. The included studies encompassed various strategies, such as creating educational resources, offering continuing education, developing public awareness campaigns, and collaborating within the community (32, 34, 40–42).

Several studies (29, 32, 34, 40, 42) pointed out that education enabled volunteers to foster community care and provide support to patients and their families, particularly in areas facing a shortage of staff. A study (42) conducted in Canada indicated that patients and their families were satisfied with the services provided by trained volunteers. They demonstrated professionalism while still maintaining an accessible and approachable demeanour. The training was designed to prepare volunteers to ensure they have the necessary knowledge and skills (32–34, 36, 38–43). During home visits, volunteers commonly engaged in activities such as assisting individuals with eating, taking medication, moving, listening, communicating, and providing information (35, 37, 40–42). Educational resource availability can vary across diverse areas. For example, a descriptive comparative study (38) revealed similar opportunities for basic and continuing training among volunteers in several European countries, with some settings offering advanced courses. Thus, the available support and education for volunteers varied depending on their regional commitment.

#### 3.2.2 Theme 2: factors related to developing community volunteers

Of the reviewed studies, nine (30–32, 35, 36, 39, 41–43) indicated factors associated with volunteers' capacity to perform their roles, which mainly involved personal experiences, training, and receiving support. While demographic information was collected in the included studies, only one (43) specifically investigated the correlation between participants' demographics (i.e., age, gender, education, and religion) and their competence after training. The findings of this study indicate that there was no significant relationship between these factors.

##### 3.2.2.1 Personal experiences

The experiences of volunteers are mentioned in three studies (35, 41, 42). The findings provide an understanding of the motivating factors and actions of individuals. For instance, Söderhamn et al. (35) found that positive feedback motivates volunteers to sustain their involvement. Furthermore, they also found that personal experiences, such as having a family member receiving palliative care, can contribute to an individual's understanding of patients in similar situations. Lee and Lee (41) point out that volunteering brings fulfilment and satisfaction and boosts self-esteem, although in some instances, volunteers experienced difficulty in bonding with patients and their families. However, the personal factors and experiences encountered were uncontrollable variables.

##### 3.2.2.2 Resource and support

Education plays a pivotal role in enhancing the knowledge and skills of volunteers in the reviewed literature. Organised training programmes with appropriate and clear content (30, 31, 35, 39), interactive activities (30–32, 39, 43), and continuous support (35, 42, 43) all contributed to this process. Two studies (30, 31) emphasised the advantages of presenting content in a straightforward manner. Focusing on the main point makes information more accessible and easier to understand for learners. Söderhamn et al. (35) highlight the significance of clarifying information on volunteers' responsibilities under legislation when providing care.

The effectiveness of interactive activities is demonstrated in three of the studies (32, 39, 43). Activities such as discussions (30–32, 39, 43), role-play sessions (32, 39, 43), case studies (32, 43), and hands-on exercises (32, 39, 43) encourage volunteers to apply their newly acquired knowledge and skills through practical scenarios. Online courses (31, 39) highlight the challenges involved in conducting educational programmes in virtual settings. Moton et al. (39) discovered that although participants were satisfied with the online sessions, such as videos, which allowed them to review materials at their preferred times, they encountered difficulties grasping some content. Bollig et al. (31) reveal that instructors often face technical problems and lack interactivity, typically in traditional in-class settings. The absence of face-to-face interaction could make it more difficult to actively engage with the course material and discussions.

Ongoing support and mentoring are practical approaches that enhance the competencies of volunteers. While training prepares volunteers with the basics, it is essential to consider the potential challenges. An initial challenge volunteers face is managing families' expectations and gaining a clear understanding of their tasks and responsibilities (35, 42). Six authors (32, 34, 35, 40, 42, 43) mentioned methods of supporting volunteers, including mentorship and access to suitable resources. Three studies (35, 42, 43) demonstrated that volunteers who receive constructive criticism and guidance through mentoring programmes could improve their skills, thus become more effective. Additionally, financial support is mentioned in the facilitation of volunteer palliative care training. Spice et al. (36) revealed that assisting with covering training session registration fees increased opportunities for volunteer palliative care education.

#### 3.2.3 Theme 3: main topics and training contents

Of the selected studies, 14 (29–35, 37–39, 41–44) mentioned learning content proposed for volunteers, which would provide palliative care in the community or in patients' homes; 11 studies (29–31, 34, 35, 37–39, 41, 42, 44) pointed out the general training programmes offered for palliative care, and three studies indicated training specific to terminally ill patients (32, 33, 43). Most training programmes (29–32, 39, 41–44) were designed based on literature reviews and content experts, with different objectives and necessities for each context. For example, Wang et al. (43) developed a training programme for community volunteers working with end-of-life patients; thus, the curriculum emphasised specific care for providing end-of-life care. Three studies (29–31) originated from the same curriculum resource, but no studies emphasised specific paediatric palliative care training. Only one study (44) took the form of a training framework that focused on palliative care for children. Two main themes emerged to delineate the primary content categories suggested for volunteers: (1) Establishing networks to sustain care; and (2) Crucial palliative care content.

##### 3.2.3.1 Establishing networks to sustain care

As a community volunteer, establishing networks to sustain care is a primary domain of necessary personal skill development for assisting in palliative care delivery. A preparatory session is recommended to comprehend the requirements involved. The scope of volunteer preparation, such as orientation (29, 31, 37, 39, 41) and ethics of care (29, 31, 35, 37), was articulated in several studies. Cultivating positive attitudes (33, 43) towards caring for patients receiving palliative care and reflecting on the significance of self-care in addition to promoting the wellbeing of others (33, 37, 39, 41, 43, 44) were identified as the main topics of expected learning.

Roles and responsibilities (29–35, 37–39, 41–44) were indicated in all of the studies to help learners understand what they can do to care for patients. Moreover, training programmes illustrated the required level(s) of competency for working with others (34, 39, 42, 44) to coordinate and deliver care in both healthcare units and patients' homes. Prince et al. (34) developed a palliative care model with a focus on educational needs through events (i.e., health fairs and public meetings) and cultural knowledge sharing to foster skills and competence and improve awareness among community members. These approaches are designed to encourage community engagement in creating compassionate communities by building support networks, a curricular topic mentioned in three studies (29–31), which was based on the Last Aid Course.

##### 3.2.3.2 Crucial palliative care content

Palliative care principles and practices (30, 32–34, 37, 39, 41) were the main topics of knowledge and understanding in providing care for patients. The learning content focuses on fundamental understanding and providing holistic care skills to alleviate the suffering of patients and their families (29–34, 37, 41, 43). In particular, different knowledge and skills are required for specific patient groups (37–39). For example, Morton et al. (39) developed a palliative care curriculum topic by integrating the role of religion in cancer patients. Furthermore, a study in Belgium (37) revealed that healthcare organisations offer more training than others to community volunteers in palliative care-trained subjects related to more specific patient groups, such as dementia or cancer. Thus, in paediatric palliative care, well-defined areas of specialisation assume paramount importance.

Several studies indicated comprehensive guidance about crucial elements of end-of-life care (29–34, 37, 41, 43), including understanding death and dying, and bereavement (30–35, 41, 43). A crucial aspect of delivering compassionate care to patients and their families during challenging circumstances is recognising and respecting their cultural practices and religious beliefs (29–34). Volunteers are expected to demonstrate effective communication skills (29–35, 38, 39, 41, 43, 44) that go beyond simply navigating the healthcare system and providing information about available resources and services. Four studies (32–34, 39) highlighted the importance of training in non-verbal communication and culturally appropriate conversations. This ability is crucial for supporting patients and their families during difficult circumstances, ultimately establishing a supportive environment and improving the quality of care provided in palliative care settings.

#### 3.2.4 Theme 4: training models for community volunteers

Delivery models and training designs varied across different settings. Of the 16 studies, eight (30, 32–34, 40–43) were conducted through face-to-face sessions, while one study (31) was administered online. Only one study (39) employed hybrid methods, such as video conferencing, online video content, and hands-on training at the site. Regarding training duration, seven studies (30–32, 40–43) focused on providing short-term training, which ranged from 1 to 3 days. For instance, Bollig et al. (30) designed a 45-min training course for each module during 1 day on the notion that individuals would prefer to attend only once, which would minimise time consumption. In contrast, two studies (33, 39) implemented teaching sessions over several weeks.

The training programme encompassed a range of teaching tools and resources, including videos (33, 34, 39), lecture slides or learning manuals (32–34, 42, 43), and pamphlets (34) or a combination of the above. Culturally appropriate materials have been emphasised in two studies (32, 34). Diverse techniques were utilised in training, with lectures and discussions predominating. Two studies (30, 31) highlighted utilising inclusive and easy-to-understand language when providing palliative care education to the general public. Furthermore, one study (43) illustrated how interactive role-playing exercises and real-life scenarios were incorporated into each programme session to ensure effective curriculum delivery. Teaching activities may be subject to time limitations imposed by the educational programme.

Several studies (30, 31, 34, 40, 42) highlighted the qualifications of instructors such as certified trainers, local healthcare providers, or palliative care preceptors. Practical knowledge of topics such as death and dying is essential to gain an understanding of a culturally sensitive and respectful approach to care. A Canadian study (34) pointed out that healthcare professionals may not necessarily provide this insight; however, knowledge shared by community elders who have in-depth understanding of relevant traditions and practices might be more effective. Three studies (40, 42, 43) revealed post-training monitoring to ensure volunteers possessed the necessary skills and knowledge to perform their roles. For instance, one study (40) conducted in Australia reported mentors who maintained contact with volunteers on a weekly basis to address any emerging concerns.

The studies included in this review used several methods and models of delivery. The evaluation of training programme effectiveness in relation to measuring knowledge and skills (33, 39, 40, 43), self-care awareness (43), self-efficacy (42), satisfaction (39, 41, 42), competency in coping (33, 41), and feedback for the curriculum (30–32, 39). For example, Pesut et al. (42) monitored post-training and evaluated the programme at 6 and 12 months using self-efficacy and satisfaction questionnaires. However, none of the studies implemented and evaluated training specific to palliative care for children. Only one study (44), which included the training framework, proposed a flexible approach to curriculum development, depending on the educationist.

## 4 Discussion

### 4.1 Key findings

This study explored the overview of palliative care training for community volunteers. Using a scoping review method (24, 25), 16 studies were examined, covering diverse study designs for which mixed methods were the primary approach. The studies revealed the importance of developing volunteer training programmes to enhance the context of palliative care as well as understanding the engagement of volunteers. While volunteer training in adult palliative care research has been extensive, there has been a lack of emphasis on paediatric palliative care. This may be due to the higher proportion of adults requiring palliative care, which has shaped most training programmes, and associated research, towards adult needs. Volunteers are often seen as an informal, non-medical workforce, and their role is often not recognised. Additionally, the complexity of interacting with children at different developmental stages can make designing effective training programmes for volunteer roles in paediatric care difficult. The research team are currently undertaking the recognised need for further research to explore volunteer roles and the development of appropriate training provision.

Many of the studies investigated education at the community level for non-healthcare professionals in providing palliative care to individuals. Community-based participatory research has been utilised to develop and implement interventions based on a community's needs (32, 34, 36). Prince et al. (34) conducted participatory action research over a 6-year period by incorporating community perspectives and knowledge. They aimed to understand the needs and unique cultural and social aspects and develop practical approaches in palliative care that shape a community's experiences. Therefore, it is not surprising that the design of the training varies depending on the setting. Different health systems have implications for volunteer training programmes. Each country has its own set of regulations and standards for volunteer training (38), which may impact the delivery methods utilised. Moreover, education and training are not limited to volunteers who work in palliative care but are used to educate and increase public awareness of palliative care to build compassionate communities (29–32, 34).

To establish an effective educational programme, it is essential to consider the factors that can help improve learner competency. Certain personal characteristics or experiences may inspire individuals to become volunteers in this field. For example, those who have first-hand experience of the impact of serious illness or loss often develop the desire to support others in similar situations (35). Wang et al. (43) used motivational screening for selected participants who demonstrated low-risk factors, such as depressive symptoms, perceived stress, over-commitment, and lack of flexibility, to qualify to be an end-of-life care volunteer; in this context, participants with high-risk factors may face challenges. The authors determined that, after training, the differences in competency were insignificant, regardless of the participants' demographics. This implies that the volunteer's demographics may not play a significant role in determining their competence level after completing training. While personal factors may motivate individuals to volunteer in palliative care, educational programmes are essential for equipping them with the necessary skills and knowledge. Moreover, it also enhances attitudes and self-confidence in relation to palliative care (20).

According to a previous systematic review by Horey et al. (45), no studies that specifically examined training programmes for palliative care volunteers in community settings met the inclusion criteria. The authors identified that the included studies focused on volunteers in organisations that provided palliative care and interventions such as randomised controlled trials (RCTs), quasi-RCTs, controlled before and after, and interrupted time series. However, our review expanded the scope to include volunteers and non-professionals interested in participating in palliative care services in diverse study designs. We found that only evaluations of community-based volunteer empowerment programmes have been published in recent years, and no studies are specific to caring for children. Similarly, a systematic review by Li et al. (20) revealed that the available curriculum was designed to instruct healthcare professionals in paediatric palliative care, but volunteer training remains a common practice in general. To bolster the delivery of palliative care for children, it is important to further study and refine volunteer training initiatives, thereby enabling volunteers to serve paediatric patients more effectively.

Although the included studies found only one study (44) proposed an educational framework emphasising paediatric palliative care education at the public health level, further studies are needed on evaluation and implementation. The training and mentoring offered aimed to enhance volunteers' skills and knowledge so they could carry out their responsibilities in palliative care. The content relied on their roles and activities in palliative care provision, communication skills, emotional support, symptom management, understanding end-of-life care, cultural sensitivity, and maintaining appropriate boundaries. While participants were prepared before volunteering, they reported a lack of clarity regarding tasks, particularly when they began providing services (35, 42). This confusion may have been due to divergent expectations when establishing a rapport with patients and their families. In some cases, families expected more than the volunteers were trained to provide. These mismatches present challenges in designing training and developing interventions that are responsive to family needs and mindful of the limitations of volunteer capacity. Thus, defining the roles, understanding provisions in palliative care, and understanding the details of tasks are essential to preparing volunteers.

Finally, this review found various curriculum designs, but most have a main emphasis on improving learners' knowledge and attitudes. Through interactive sessions, case studies, and discussions, participants can recognise other viewpoints and develop their ideas to foster a more compassionate and inclusive approach to care (30–32, 39, 43). Moreover, education and training are not limited to the classroom but extended to community workshops, events, or public forums to educate and increase public awareness of palliative care (34). Bollig et al. (31) proposed an online training course, which emerged as a helpful tool for addressing the limitations imposed by face-to-face classrooms during the pandemic. It could offer several advantages, including enhanced accessibility and flexibility for broader participants. However, it may be insufficient to provide cultural knowledge and practice in the context of community-based care, as participants come from diverse settings. Accordingly, cultural aspects should be considered in the development and delivery of palliative care training, which may require additional time for sharing and discussion.

### 4.2 What this study adds?

This review clarifies the existing evidence on paediatric palliative care training for community volunteers, offering valuable insights into its development, factors, and designs. Studies on public health approaches to palliative care are increasingly mentioned, with an aim to enhance community-based care through collaboration between healthcare providers and non-healthcare providers in addressing palliative care issues. A public health approach would be beneficial in providing assistance to carers who experience psychological distress and who may require longer-term support (46). A systematic review suggested that the public health approach to palliative care influences a transformation in terms of practicability, strengthens community development, and changes attitudes towards those facing terminal illness (47). Therefore, public health education initiatives on palliative care are imperative.

The concept of public health approach to palliative care has garnered significant attention in recent years (11–13), reflecting a shift towards more holistic and community-oriented approaches to healthcare. Aoun et al. (48), for example, emphasised the importance of trained volunteers and demonstrated the value of gaining knowledge and skills while also enhancing their connections to the community and building networks of support. Although there has been a growing recognition of public health palliative care education, training specifically concerning palliative care for children has not yet been widely disseminated. The review highlights the lack of empirical research into training volunteers caring for children and young people at the end of life.

Additionally, we found the challenges volunteers encountered, even if they felt well-prepared. This will be useful in recognising these obstacles and finding the appropriate method to improve effective training. Evaluating the effectiveness of training is challenging in this field and requires careful consideration, because each setting has a unique cultural and practical approach tailored to suit its needs. Cultural beliefs in particular play a crucial role in shaping practical approaches, which can impact the success of the training design. Each setting has its own considerations, such as time, resources, and support. Thus, training design can be tailored to accommodate these factors to ensure success. Understanding palliative care therefore is not, merely, abstractly conceptualised, but should be co-designed and applied in practice through public health perspectives (49, 50). Incorporating community input will be essential to the development of the training, which will gain insights into their perceptions, needs, and expectations regarding palliative care.

As palliative care relies increasingly on informal workforces, such as volunteers, this evidence is crucial to shaping educational developments. Mapping the existing evidence in palliative care training for community volunteers revealed that, while progress has been made, significant gaps remain in paediatric care. These gaps provide a starting point for future research that will develop and enhance the training of volunteers who provide care and support to paediatric patients within the community.

### 4.3 Strengths and limitations of the review

To our knowledge, this paper is the first to scope the literature around palliative care training for community volunteers. This scoping review employed a rigorous and transparent methodology, ensuring the integrity of the results. We conducted an independent screen with at least two reviewers to minimise bias in the process of selecting articles for this study. Our review encompassed research from various countries and cultural backgrounds to offer essential evidence that will guide the design of future training programmes.

Although a systematic process was undertaken to identify and search for articles, some evidence might possibly have been missed that exists in the grey literature. We excluded non-English language literature and may, therefore, have missed out on potentially relevant studies in other languages. The search was limited to six databases and a hand search of bibliographies. We sought published studies but did not exclude the literature and research protocol due to quality concerns; we were keen to include a diverse array of literature encompassing this field. The review and discussion rely on the details presented by the authors in the reviewed literature, with limited generalisability due to specific contexts in education available in the community.

## 5 Conclusion

This scoping review found that community-based palliative care approaches recognise the importance of involving non-healthcare professionals in providing care and support to individuals with life-limiting illnesses. The role of community volunteers has become increasingly recognized, sparking interest in their training and education in recent years. Providing volunteers with the necessary training and resources is an essential cornerstone of enhancing palliative care at the public health level. Most of the studies reviewed focused on palliative care among adult patients. We must expand our understanding of training needs specific to paediatric palliative care. The training design might incorporate interactive activities that promote cross-cultural understanding and collaboration with communities in development and delivery. Importantly, educationists should tailor their training design to optimise the practicality of local needs by considering the cultural aspects and practical approach of each setting and promoting interprofessional collaboration and person-centred care, which will equip them to provide effective palliative care and support a valuable workforce. The findings of the review have practical implications that will be useful to researchers, public managers, and wider society to inform future education for the volunteer workforce specific to palliative care. In particular, future research and evaluation should focus on children palliative care education and interventions to better support volunteers caring for children and young people with serious illness and palliative care needs.

## Data Availability

The original contributions presented in the study are included in the article/Supplementary material, further inquiries can be directed to the corresponding author.
