# Supplementary material for: Palliative care training programmes for community volunteers working with children and their families: a scoping review
Source: Front Public Health. 2025 May 16;13:1469854. doi: 10.3389/fpubh.2025.1469854 (PMC12122434; doi:10.3389/fpubh.2025.1469854)
Supplement: Supplementary file 1 [file Data_Sheet_1.pdf]

frontiers

Supplementary Material S1

MEDLINE search strategy

EBSCOhost

Saturday, January 13, 2024 9:31:53 AM

#	Query	Limiters/Expanders	Last Run Via	Results
S1	AB (Volunteer* or "lay people" or "ordinary people" or non- professional* or civic or citizen* or public) AND TI (“P?ediatric palliative care” or "children's palliative care" or "palliative care" or supportive care or "end of life care" or "death literacy" or "death and dying" or life-limiting or life-threatening) AND AB (training program* or education* or “training course*” or curriculum* or module* or teaching) AND AB (Communit* or community-based or "compassionate communit*" or "health- promoting palliative care" or "public health" or rural)	Limiters - Publication Date: 20000101-20231231; English Language<br>Expanders - Apply equivalent subjects<br>Search modes - Boolean/Phrase	Interface - EBSCOhost Research Databases<br>Search Screen - Advanced Search<br>Database - MEDLINE	184
