# Supplementary material for: Palliative care training programmes for community volunteers working with children and their families: a scoping review
Source: Front Public Health. 2025 May 16;13:1469854. doi: 10.3389/fpubh.2025.1469854 (PMC12122434; doi:10.3389/fpubh.2025.1469854)
Supplement: Supplementary file 2 [file Data_Sheet_2.pdf]

Supplementary Material S2

Table 2: Summarised characteristics of included studies

**Author/year/ study location**	**Study type**	**Aim**	**Study populations**	**Methodology and methods**	**Important results**
Aoun et al., 2020, Australia	Study protocol (quasi experiment al design)<br><br>- data collection spanning 10 months.	1) To design and implement a volunteer-led model<br><br>2) To evaluate a training programme for volunteers<br><br>3) To assess the feasibility of the model	Participants include four groups: patients, family carers, the connectors and caring helpers.	1) Train connectors to work with palliative care families.<br><br>2) Measure pre- and post-intervention using a questionnaire<br><br>3) Interviews and focus groups with connectors, caring helpers and palliative care providers.	- This model was established for community palliative care services by training the volunteers called connectors to co-design a plan with families.<br><br>- Establishing a 2-day training course and developing training resources to assist volunteers in understanding their role, staying informed about the community, and preparing to manage situations.
Bollig et al., 2019, Publications regarding Last Aid courses	Review Article	To provide an overview on the Last Aid course concept and the compassionate community approach	Last Aid course participants (nonprofessio nals)	Review of the publications regarding Last Aid courses using search engines PubMed and Medline. Additional sources were searched in books, reference lists, and the Internet.	- The curriculum aimed at educating nonprofessionals on providing support for palliative care. The course spans four modules in a single day (each lasting 45 minutes) with the following themes: care at the end of life, advance care planning and decision-making, symptom management, and cultural aspects of death and bereavement.

Author/year/ study location	Study type	Aim	Study populations	Methodology and methods	Important results
Bollig, Kristensen and Wolff, 2021, Germany, Switzerland and Austria	A mixed methods design	1) To test the feasibility and acceptability in different countries<br><br>2) To explore the views and experiences of participants with the course.	5,469 Last Aid course participants	Questionnaire to evaluate the course, including both quantitative and qualitative data	- The four modules were taught for one day.<br><br>- 76% of participants rated the course very good, while the lowest-ranked modules related to communication and decision-making.<br><br>- The factors supporting learning were a nice atmosphere and using case stories.<br><br>- The challenges were time and opportunities in discussions.
Bollig et al., 2021, Germany and Brazil	A mixed methods design (pilot study)	1) To test the feasibility of online courses<br><br>2) To explore the experiences of participants and instructors with the online course.	92 participants in 15 online Last Aid courses	Questionnaires to evaluate the online course and an online focus group discussion with course instructors.	- The online course consists of 4 modules, each 45 minutes.<br><br>- 80% of the participants rated this course as very good. However, they reported limited opportunities to engage in discussions in online courses. Participants prefer face-to-face in a classroom, which is helpful for discussion.
Chou et al., 2008, United States	Action research	1) To describe the development of CACCC	Chinese-American community	1) A needs assessment was conducted using focus group discussions with Chinese Americans and interviews	- The course material was divided into four 90-minute modules: 1) introduction to end-of-life care, 2) end-of-life spiritual

Author/year/ study location	Study type	Aim	Study populations	Methodology and methods	Important results
		2) To evaluate one of CACCC's first public programs, a volunteer and caregiver<br><br>3) To report on CACCC's current projects and goals.<br><br>4) To discuss the implications	(82 Chinese Americans, 8 Chinese-speaking physicians, 99 trainees)	with Chinese-speaking physicians.<br><br>2) Establish a working group to train volunteers and caregivers and create a resource for the Chinese-speaking population.<br><br>3) Providing training for healthcare professionals and the public.	issues, 3) guide to patient care, and 4) end-of-life psychosocial issues.<br><br>- Most participants cited the role-playing exercises and sharing of personal experiences, the presentation on language and communication, the introduction to spiritual issues, and the discussion of cultural diversity as the most valuable components of the training. Volunteers who had limited patient care experience preferred interactive formats and simple, practical instructional materials.
Claxton-Oldfield, Crain and Claxton-Oldfield, 2007, Canada	Quantitative research designs<br><br>(quasi experimental design)	To examine the effects of palliative care volunteer training on individuals' levels of death anxiety and death competency.	12 volunteers and 5 home support workers	- Pre-post training questionnaires consisted of the fear of death scale, coping with death scale, and a demographic information questionnaire.	- The training programme consisted of nine sessions over nine weeks (27 hours in a total)<br><br>- The core topics covered in training typically include the definition of palliative care, the palliative care team/roles, signs and stages of death and dying, spirituality, grief and bereavement, and communication.<br><br>- The results indicated that the coping with death scale was improved, while the Fear of Death Scale scores did not substantially change after training.

**Author/year/ study location**	**Study type**	**Aim**	**Study populations**	**Methodology and methods**	**Important results**
Lee and Lee, 2020, Korea	A sequential mixed methods design	1) To assess the feasibility and effectiveness of a volunteer program<br><br>2) To assess the caregiver's satisfaction with the services.	- 19 volunteers<br><br>- 5 patients and 5 caregivers	Pre- and post-training and after completing the volunteering period using questionnaires (death anxiety scale, coping with death scale, and meaning-in-life scale).<br><br>- Survey volunteers' satisfaction with training and caregivers' satisfaction with volunteer service.<br><br>- Focus group with volunteers	- The training programme consisted of 12 sessions in two days (16 hours).<br><br>- The findings revealed that volunteers scored high on confidence in performing activities and satisfaction with the training.<br><br>- The most common activities were emotional support and spiritual support.<br><br>- Several volunteers faced challenges in communicating with patients and their families initially.
Monton et al., 2023, United States	A mixed methods design	To assess the knowledge, perceived competence, and satisfaction of community health workers with the training	- three community health workers<br><br>(community volunteer workers)	- Pre-training and post-training questionnaires to assess knowledge, perceived competence, perceived preparedness and a post-training survey on satisfaction with the training<br><br>-Interviews with community health workers the week following the completion of training	- Training developed through collaboration from community health workers and experts with content from reputable organisations and external sources. The curriculum was delivered over 3 months using a blended learning approach:<br><br>1. Synchronous training by video conferencing over 1 week (around 30–60 minutes per session).<br><br>2. Asynchronous training via weekly modules over 4 weeks by learning the

Author/year/ study location	Study type	Aim	Study populations	Methodology and methods	Important results
					content online and having a group discussion.<br><br>3) Experiential training<br><br>After training, participants' perceived competence score and all knowledge test scores have increased. Overall, participant feedback indicated that they were satisfied with the training. They suggested that problem-based learning sessions would be useful once they began working with patients.
Neilson et al., 2021, United Kingdom	Action research	To develop a children's palliative care education and training standard framework	35 participants<br><br>(Institutes of Higher Education, clinical practice or 3rd sector allied organisation)	- Focus groups (World Café style) conducted over 4 meetings to discuss the need to standardise children's palliative care learning and develop and revise the education framework	The education framework has developed particularly in palliative care for children, which has four expected levels of learning outcomes: public health, universal, core, and specialist. The public health level provides guidance on course design and content to educationalists to address palliative care in public health issues while allowing flexibility in delivery and assessment.
Pesut et al., 2018, Canada	A mixed methods design	To assess the model of volunteer navigation in the early palliative	- 7 volunteers<br><br>- 18 patients	- Volunteers received training and visited patients every 2-3 weeks over 12 months.	- Volunteers were supported by a nurse navigator who provided oversight and mentoring.

Author/year/ study location	Study type	Aim	Study populations	Methodology and methods	Important results
		phase for adults living in community.	- 1 nurse and 2 stakeholders	- Evaluated using questionnaires of self-efficacy and satisfaction, quality of life, and perceptions of the programme.<br><br>- Interview	- Training in a three-day workshop and curriculum, which included learning manuals, case studies, and workshop materials, was developed to prepare volunteer navigators using evidence-based support.<br><br>- Volunteers felt well-prepared and satisfied with their competence; however, the lack of clarity regarding their role posed a significant challenge for volunteers to provide care for patients effectively.
Prince et al., 2019, Canada	Action research	To assess and address local palliative care educational needs to improve community capacity in palliative care.	185 participants<br><br>(community members, elder and knowledge carriers, internal health care providers, and external health care providers)	Phase 1: Focus groups, interviews, and surveys to assess community needs<br><br>Phase 2: Develop an action guide for the palliative programme. Phase 3: Identify educational resources and implement	- Community members lacked palliative care knowledge, insufficiently trained staff, and a lack of training offered to volunteers within the community.<br><br>- Training needs were identified regarding cultural knowledge and practices.<br><br>- Education and training were implemented through multiple strategies, including engaging elders and knowledge carriers to share cultural knowledge and model culturally appropriate conversations about death and dying at workshops, community meetings or events, providing

Author/year/ study location	Study type	Aim	Study populations	Methodology and methods	Important results
					bedside education and creating culturally relevant resources.
Söderhamn et al., 2017, Norway	Qualitative research designs<br><br>(a descriptive phenomen ological study)	To describe volunteers' lived experiences as volunteers in palliative care within the community health care services.	9 volunteers	- Using individual interviews conducted with volunteers in palliative care.	- Volunteers attended a training course led by a palliative care coordinator.<br><br>- Experience emerged in six features:<br><br>(1) A privilege being a volunteer: positive feedback was meaningful in motivating and encouraging them to continue their volunteering.<br><br>(2) To be present: having attention time to support<br><br>(3) To face and deal with challenges: facing a painful experience when patients die/ get worse or emotionally challenged.<br><br>(4) Knowledge and life experience: training communication skills were necessary for volunteers.<br><br>(5) A clarified role: role clarity was essential to service by volunteers.<br><br>(6) To be followed-up: continuously by a mentor. Volunteers highly appreciated

**Author/year/ study location**	**Study type**	**Aim**	**Study populations**	**Methodology and methods**	**Important results**
					being trained and supported by a skilled mentor.
Spice et al., 2012, Canada	Action research	To explain the development, implementation and achievements of the rural palliative care programme	Interdisciplinary team	1) Development phase: to identify needs and gaps in rural palliative care service delivery using survey and focus groups.<br><br>2) Implement the programme regarding needs and follow-up feedback through focus groups.	Addressing the top six priorities<br><br>1) Education for staff<br><br>2) Linkages the resources and improve access to specialists<br><br>3) Psychosocial support for patients and families<br><br>4) Facilitate rural home deaths<br><br>5) Education and utilization of volunteers: using competency-based palliative care and bereavement education programmes for volunteers by the Calgary organisation and covering the registered fee.<br><br>6) A mobile specialist consultation team
Vanderstichelen et al., 2018, Belgium	Quantitative research designs<br><br>(Survey)	To describe organised volunteerism in palliative direct patient care.	342 healthcare organisations providing care for people with	Postal survey by using a self-developed questionnaire	- All community home care organisations had volunteers providing psychosocial care for patients and their relatives.<br><br>- Volunteer training is offered in community settings (100%), and the most common training topics (>80%) were

Author/year/ study location	Study type	Aim	Study populations	Methodology and methods	Important results
			terminal illnesses		themes regarding specific patient groups, ethics, lifting techniques, guarding personal and professional boundaries, and volunteers' needs. The fewer training topics offered related to advanced care planning.<br><br>- Obligatory training was offered at almost 30%.
Wang et al. 2020, Hong Kong	Mixed methods design (quantitati ve dominant)	To examine the effectiveness of a holistic capacity-building program for volunteers in community-based end-of-life care.	171 participants (to be community volunteers)	- interview (motivational screening)<br><br>- A single-group longitudinal design with a pre-training test, post-training test, and 6-month follow-up test	- The training curriculum comprises four sessions (16 hr.) on 8 core competencies in end-of-life care, which took formats of mini-lecture, role-play, case sharing, and coached discussion during face-to-face classes. -The competence in end-of-life care, awareness of self-care, and death work competence significantly improved after training and remained intact at 6-month follow-up.
Woitha et al., 2014, Belgium France, Germany, the Netherlands, Poland,	A descriptive study	To explain an overview of volunteer work in palliative care in 7 European countries.	- volunteer organizations in palliative care<br><br>- experts (n = 6–12, per country	A literature search combined with an interview study.<br><br>- comparison of data	The overall involvement of volunteers in palliative care across seven countries is different. The established training course varies according to roles and settings. All countries indicated they offered initial and continuous training for volunteers, particularly Germany, which offered

Author/year/ study location	Study type	Aim	Study populations	Methodology and methods	Important results
Spain, and England					advanced courses emphasising knowledge and skills for practice work.
